# Ischemic Stroke Outcome Predicted by Serum Albumin Levels

**DOI:** 10.7759/cureus.59816

**Published:** 2024-05-07

**Authors:** Payod Kumar Jena, Tanmaya Padhy

**Affiliations:** 1 Department of Neurology, Sunshine Hospital, Bhubaneswar, IND

**Keywords:** ischemic stroke, blood clot, thrombolytic therapy, serum albumin, atherosclerotic plaque

## Abstract

Objective and aim: This research aims to assess the predictive importance of serum albumin levels in individuals who have recently experienced an acute ischemic stroke and to establish a correlation between these two variables.

Materials and methods: A prospective hospital-based investigation with 50 participants was conducted after receiving ethical approval from Sunshine Hospital, Hyderabad, India. Patients older than 18 years old who had radiological or clinical evidence of having suffered an acute ischemic stroke were considered for participation in the research.

Results: Albumin levels in the blood are typically about 3.6 g/dL. One patient between the ages of 46 and 55 had low serum albumin levels. Many people in both groups had albumin levels of about 4.4. Serum albumin concentration was measured using the modified Rankin Scale (mRS). After one week and three months, 32 patients had mRS values of less than three, whereas 16 had mRS values of greater than three-one; the individual presented with an mRS value over 3, as well as a blood albumin level below 3.5. The p-value ended up being 0.428. No link could be supported by the statistical evidence identified. P = 0.249 indicated no association between serum albumin and National Institutes of Health Stroke Scale (NIHSS) scores. According to the findings of this inquiry, there is no correlation between the amounts of albumin in the blood and the NIHSS scores.

Conclusion: This study did not find a correlation between higher blood albumin levels and a worse outcome after an ischemic stroke. It contradicts the corpus of current knowledge.

## Introduction

Ischemic strokes are the predominant of all stroke cases, accounting for over 87% [1]. Ischemic stroke occurs when there is a blockage in a blood artery that delivers blood to the brain caused by an atherosclerotic plaque or clot [1]. Within a short period, brain cells perish due to this obstruction, resulting in the deprivation of essential nutrients and oxygen to the brain. The clinical consequences of an ischemic stroke can lead to a range of severe physical, cognitive, and emotional impairments, including paralysis, speech difficulties, memory loss, and emotional disturbances [2,3]. Despite the advancements in thrombolytic therapy and endovascular methods for stroke management, accurately predicting the long-term effects of an ischemic stroke remains challenging. Early identification of patients at higher risk of adverse outcomes is crucial for optimizing treatment and rehabilitation strategies, potentially leading to enhanced quality of life for stroke survivors.

The liver synthesizes serum albumin, a protein circulating in the bloodstream [4], which helps maintain oncotic pressure. Furthermore, it is critical for preserving cell membranes and is an essential antioxidant [5]. Serum albumin has garnered attention due to its potential as a biomarker for several diseases and health conditions, as well as its role in maintaining overall health. Recent research indicates a likely correlation between serum albumin levels and the prognosis of ischemic stroke. Research is underway to ascertain the ability of serum albumin to forecast the prognosis of stroke patients effectively. Serum albumin serves as an indicator of both inflammation and nutritional status. Reduced blood albumin levels are associated with poor dietary status and chronic inflammation, and these factors may affect the outcome of stroke [5-7].

Malnutrition and inflammation following a stroke might lead to a weakened immune system and a higher susceptibility to infections [8]. Furthermore, studies have demonstrated a correlation between a decreased amount of serum albumin following an ischemic stroke and a heightened susceptibility to complications such as infection, cerebral edema, and hemorrhagic transformation [9]. The presence of these complications is strongly associated with adverse outcomes, including increased mortality and long-lasting impairment. Furthermore, these complications have the potential to exacerbate the initial damage caused by the stroke [10]. Serum albumin can indicate overall health and the presence of concurrent conditions, such as diabetes, renal disease, and cardiovascular disease [9,10]. The correlation between blood albumin levels and prognosis may be attributed to the acknowledged impact of these comorbidities on ischemic stroke outcomes.

The fields of neurology and medicine attach significant significance to comprehending and predicting the outcomes of ischemic stroke. The role of serum albumin as a prognostic biomarker in this context is an emerging field of study that could yield valuable insights into the post-stroke prognoses of patients. Scientists want to enhance our capacity to detect individuals at elevated risk, customize treatment approaches, and ultimately improve the quality of life for those affected by ischemic stroke by investigating the correlation between serum albumin levels and stroke outcomes. Further study and extensive clinical investigations are necessary to assess the therapeutic efficacy of serum albumin levels in predicting the outcomes of ischemic strokes and determining their suitability for inclusion in standard stroke therapy. The aim of this study was to evaluate the prognostic significance of serum albumin levels in people who have recently suffered from an acute ischemic stroke.

## Materials and methods

This study is a hospital-based examination that involves 50 people and follows a prospective approach. We initiated this investigation after obtaining ethical approval from the institution under reference (IUN/2256/18). The study encompassed all patients aged 18 years or older who exhibited radiological or clinical evidence of an acute ischemic stroke. The exclusion criteria included any factors that could potentially cause cerebral hemorrhage, liver disease (excessive alcohol consumption of more than 20 units per week for males and more than 14 units per week for women), heart failure, nephrotic syndrome/diabetic nephropathy, protein-losing enteropathies, malignancies, and conditions that mimic stroke.

Skilled research coordinators recorded baseline data during admission. We collected information on patients' demographics, medical histories, initial diagnoses, acute recanalization therapy, prescriptions given during hospital stays, and outcomes of laboratory tests. We obtained the National Institutes of Health Stroke Scale (NIHSS) scores and the pre-stroke modified Rankin Scale (mRS) through in-person interviews as additional components. We used the Trial of Org. 10172 in Acute Stroke Treatment (TOAST) criteria to classify the etiology. Two neurologists scrutinized the Digital Imaging and Communications in Medicine (DICOM)-formatted images stored on CDs. We classified any artery inside or outside the brain as having either intracranial or extracranial vascular stenosis if it was 50%-99% narrowed or completely blocked. Within 24 hours of admission, researchers performed serum albumin and alanine aminotransferase (ALT) assays.

We used either the bromocresol purple assay or the bromocresol green assay to determine the serum albumin levels, depending on the test reagent. The hospital's central laboratory conducted tests for high-sensitivity C-reactive protein (hs-CRP), total cholesterol (TC), low-density lipoprotein (LDL), high-density lipoprotein (HDL), triglycerides (TG), and serum creatinine. Upon admission, we collected blood samples and refrigerated them in cryotubes at -80°C for storage. We then moved them to the central laboratory at a regulated temperature. We calculated the eGFR using the formulas published by the Chronic Kidney Disease Epidemiology Collaboration. Mortality and functional outcomes were the clinical outcomes evaluated in this investigation. We used in-person interviews to gauge these results at three- and one-year intervals. An mRS score of 3-6 indicated a disability or mortality, considered a poor functional outcome. On the other hand, an independent mRS score of 0-2 was considered a favorable functional outcome. The term "mortality" included deaths from all sources.

Statistical analysis

Microsoft Excel was utilized to manage and organize all patient data. The tables contain information on averages, medians, and ranges. The data were assessed using chi-square and ANOVA. The chi-square test was employed to analyze categorical data, while the analysis of variance technique was utilized to evaluate continuous data. The data were analyzed using Statistical Product and Service Solutions (SPSS, version 16.0; IBM SPSS Statistics, Armonk, NY). We performed hypothesis tests on the p-values using a significance level of 5%.

## Results

The relationship between age and serum albumin level fluctuations is seen in Table 1. The blood's typical albumin content is 3.6 grams per deciliter (g/dL). Of the patients between the ages of 46 and 55, one had low blood albumin levels. A significant portion of the patient population in each group fell within the albumin range of 4 and 4.4.

**Table 1:** Variations of serum albumin levels in relation to age

Age	Serum albumin 3-3.4 g/dL	Serum albumin 3.4-3.9 g/dL	Serum albumin 4-4.4 g/dL	Serum albumin > 4.4 g/dL
26-35	0	0	0	0
36-45	0	0	4	1
46-55	2	3	5	4
56-65	1	4	8	3
>65	1	3	7	3

Serum albumin with mRS scores after one week and three months is shown in Table 2. The mRS score was used to evaluate the serum albumin level. After one week and three months, there are 30 patients with a blood albumin level of more than 3.5 and four patients with an mRS score of less than 3, while there are 16 patients with an mRS score more significant than three after one week and three months. There is just one person with a blood albumin level below 3.5 and an mRS score over 3. The p-value that was found was 0.428. Consequently, no statistically significant correlation was found.

**Table 2:** Serum albumin with NIHSS scores after one week and three months NIHSS: National Institutes of Health Stroke Scale

Serum albumin	NIHSS score ≤ 10	NIHSS score > 10
3-3.4 g/dL	0	1
3.5-3.9 g/dL	9	2
4-4.4 g/dL	22	2
> 4.5 g/dL	9	4

After one week and three months, the blood albumin level is shown, together with the National Institutes of Health Stroke Scale (NIHSS) score, in Table 3. Patients who had low NIHSS scores and high blood albumin levels when they were admitted to the hospital had a better prognosis than those who had high NIHSS scores and low albumin levels, according to the findings of many research. High-albumin, low-NIHSS individuals fared similarly well in this study. One individual had elevated NIHSS and low albumin levels. The probability value was found to be 0.249. Consequently, there was no discernible correlation between the NIHSS scores and the blood albumin levels in this investigation.

**Table 3:** Serum albumin with NIHSS scores after one week and three months

Serum albumin (g/dL)	After 1 week		After 3 months	
	≤ 3 g/dL	> 3 g/dL	≤ 3 g/dL	>3 g/dL
	0	1	1	0
≥ 3.6 g/dL	30	16	40	4

## Discussion

Albumin is crucial to multiple physiological processes occurring in the blood vessels. Erythrocyte aggregation is a key process that lowers hematocrit levels by making blood thicker in low-shear situations and stopping red blood cells from settling down when there is no flow [4]. Erythrocyte aggregation is essential for their proper functioning. Albumin exhibits exceptional antioxidant characteristics [8]. Albumin inhibits thrombus development and leukocyte adhesion in the microvasculature during a stroke's reperfusion. It demonstrates neuroprotective characteristics [6]. Albumin scavenges oxygen-free radicals and slows down the production of harmful hydroxyl radicals. Albumin effectively binds to copper ions and stops the lipid peroxidation process that depends on copper ions in the cell membrane [3]. Moreover, there is a proposition that albumin establishes a connection with lysophosphatidylcholine, leading to neuroprotective advantages [2]. Through free lysophosphatidylcholine [5], inflammation damages the vascular endothelium by increasing the number of molecules that stick to white blood cells [6]. At sufficiently elevated concentrations, it also induces apoptosis [4-8]. Given the characteristics above, we hypothesized that providing albumin after an ischemic stroke could improve long-term results. The current study included participants with a mean age of 55, suggesting a primarily middle-aged cohort. The average age of people who suffer from strokes in Western regions has decreased by a factor of 10 years [5]. This study revealed that the severity of stroke was greater in male patients in comparison to female individuals. The study identified systemic hypertension and diabetes mellitus as the main risk variables. Around 40 individuals encountered favorable outcomes, while five encountered adverse results. Patients who had negative outcomes had an average infarct volume of approximately 26.51, whereas those who had positive outcomes had an average infarct volume of around 5.59. Three months later, despite a robust association after one week, the relationship between the size of the infarction and the mRS score was no longer statistically significant. Previous research has demonstrated a link between older age and unfavorable outcomes, as well as longer hospital stays. This study presented findings that directly contradict the publications cited before. By reaching 65, all patients had an mRS score of less than three at three months. Dziedzic et al. [11] assessed a patient's condition three months after an ischemic stroke using an mRS. Throughout the third month, Dziedzic et al. assessed the functional outcomes of serum albumin levels. According to this study, patients who experienced negative results showed lower amounts of blood albumin than those with positive outcomes. Idicula et al. conducted a survey and found that people with ischemic stroke and high levels of serum albumin had lower mortality rates and better outcomes [5]. Moreover, this study has shown a strong correlation between age and serum albumin levels. The relationship between blood albumin levels and functional results has not received much research in India. As far as we know, only two studies have been undertaken on this issue in India. Each of these trials focused on studying patients who had experienced an ischemic stroke to establish a connection between blood albumin levels and short-term functional results. A recent study by Babu et al. [12] found that serum albumin has a substantial impact on both the severity and prognosis of strokes. In a later investigation by James et al. [13], researchers found a significant correlation between the levels of serum albumin and the results of rapid functional evaluations. For comparison, these studies did not evaluate blood albumin levels or functional outcomes in the third month. Most research in Western countries and India did not assess the infarct magnitude, a crucial factor in determining the outcome. Hill et al. [14] conducted a survey, which revealed that individuals with low albumin levels frequently experienced recurrences. According to the current study results, most patients with a positive outcome had decreased mRS scores, albumin levels, NIHSS scores, and infarction volume. Patients with a higher NIHSS score, a more elevated mRS, a bigger infarction volume, and increased albumin levels experienced worse outcomes. After three months, the patient, who was alone and had low serum albumin levels, also had positive results. Unlike Indicula et al.'s research [5], our study found no evidence of a decrease in blood albumin levels with increasing age. Therefore, serum albumin levels do not exclusively influence the patient's prognosis; the size of the infarction also significantly influences it. The patients with negative outcomes had an average infarct volume of around 25 cc. Additionally, we found a statistically significant relationship between the infarct size and the mRS score after one week. While most patients experienced a positive clinical outcome according to the mRS score three months following treatment, the p-value did not achieve statistical significance. Previous research has established that serum albumin is a dependable marker for predicting long-term outcomes after an ischemic stroke [6-8]. Nevertheless, these recent statistics are in direct opposition to the previous conclusions. Previous studies by Martin et al. [4] and James et al. [13] have demonstrated that the administration of human albumin in high doses within two to four hours after the commencement of a stroke has a considerable positive impact on brain swelling, the size of the damaged area, and the overall functional recovery. We conducted a clinical trial, "Albumin in Acute Ischemic Stroke," utilizing all available evidence. We administered a dosage of 2 g per kilogram of albumin (intravenous 25%) in this experiment. After 90 days, there were no noteworthy occurrences. The observation of James et al. [13] indicated a high prevalence of pulmonary edema and intracerebral hemorrhage. The study's limitations included a small sample size and a lack of individuals with low albumin levels, which made it less comparable to other studies carried out in Western nations. Further investigation is required to conclusively establish whether the injection of human albumin can improve stroke patients' clinical outcomes.

## Conclusions

In conclusion, our investigation revealed that diabetes and systemic hypertension were substantial risk factors that were associated with a disproportionately high incidence of cases overall. In contrast, elevated blood albumin levels were not associated with a worsening prognosis in patients who had suffered an ischemic stroke.
